# The recognition of HIV-1 consensus group M Gag and Nef peptide reagents in mono- and multi-clade epidemics: implications for HIV vaccine design

**DOI:** 10.1186/1742-4690-9-S2-P253

**Published:** 2012-09-13

**Authors:** L Zembe, M Tongo, E Ebong, EM Ngobe, C Williamson, W Burgers

**Affiliations:** 1 University of Cape Town, Cape Town, South Africa; 2 Division of Virology, University of Cape Town, Cape Town, South Africa; 3 Institute of Medical Research and Study of Medicinal Plants, Younde, Cameroon

## Background

The high level of genetic diversity of HIV-1 poses a major challenge for global vaccine development. Vaccines based on centralized sequences would minimize genetic distances to multiple clades and potentially maximize cross-reactivity. Whether reactivity of these centralized peptide reagents differs in mono- and multi-clade epidemic is unknown.

## Methods

In this study, full-length gag and nef gene sequences (from Cameroon, 50 and 54, respectively and South Africa, 23 and 19, respectively) were characterized. HIV-specific T-cell responses to group M consensus Gag and Nef peptide reagents were characterized at the peptide level using the IFN-γ ELISpot assay.

## Results

Viruses from Cameron exhibited a large degree of genetic diversity; all subtypes were present excluding subtype C, with CRF02_AG being dominating (49%). Contrary, sequenced viruses from South Africa were all pure subtype C viruses. Despite the greater diversity of viral clades in the Cameroonian cohort, the genetic distances to the consensus M reagents was similar for both cohorts (11% and 15% for Gag and Nef, respectively). Whilst the magnitude and breadth of responses to Con M Gag and Nef did not differ significantly between the two cohorts, there was a greater frequency of responders in the Cameroonian cohort compared to South Africans (95% versus 82%, respectively). For the Cameroonian cohort, 75/182 (41%) of the consensus M peptides were targeted, while for the South African cohort 66/182 (36%) of the peptides were targeted, with the majority being recognized in only 1-2 participants (69% and 76%, respectively). Patterns of immunodominance and targeting, however, differed dramatically between the two cohorts, with only 36% and 39% of Gag and Nef peptides commonly recognized between the two cohorts.

## Conclusion

Although similarities in total magnitude and breadth may be observed between different epidemics, patterns of immunodominance of centralized immunogens may be different which may have implications for vaccine development.

